# Invasive and non-invasive diagnostic approaches for microbiological diagnosis of hospital-acquired pneumonia

**DOI:** 10.1186/s13054-019-2348-2

**Published:** 2019-02-18

**Authors:** Otavio T. Ranzani, Tarek Senussi, Francesco Idone, Adrian Ceccato, Gianluigi Li Bassi, Miquel Ferrer, Antoni Torres

**Affiliations:** 1 Department of Pneumology, Institut Clinic de Respiratori, Hospital Clinic of Barcelona, Institut d’Investigacions Biomèdiques August Pi i Sunyer (IDIBAPS), University of Barcelona (UB), ICREA Academia award, Ciber de Enfermedades Respiratorias (Ciberes, CB06/06/0028), Barcelona, Spain; 2 Pulmonary Division, Heart Institute (InCor), Hospital das Clinicas HCFMUSP, Faculdade de Medicina, Universidade de Sao Paulo, Sao Paulo, SP Brazil; 3 Department of Surgical Sciences and Integrated Diagnostics, IRCCS AOU San Martino- IST, University of Genoa, Genoa, Italy; 4 Department of Anesthesiology and Intensive Care|, Hospital “A. Gemelli”, Catholic University of the Sacred Heart, Rome, Italy; 5 Seccion Neumologia, Hospital Nacional Prof. Alejandro Posadas, Palomar, Argentina

**Keywords:** Hospital-acquired pneumonia, Microbiological diagnosis, Diagnostic methods, Hospital infections, Bronchoalveolar lavage

## Abstract

**Background:**

Data on the methods used for microbiological diagnosis of hospital-acquired pneumonia (HAP) are mainly extrapolated from ventilator-associated pneumonia. HAP poses additional challenges for respiratory sampling, and the utility of sputum or distal sampling in HAP has not been comprehensively evaluated, particularly in HAP admitted to the ICU.

**Methods:**

We analyzed 200 patients with HAP from six ICUs in a teaching hospital in Barcelona, Spain. The respiratory sampling methods used were divided into non-invasive [sputum and endotracheal aspirate (EAT)] and invasive [fiberoptic-bronchoscopy aspirate (FBAS), and bronchoalveolar lavage (BAL)].

**Results:**

A median of three diagnostic methods were applied [range 2–4]. At least one respiratory sampling method was applied in 93% of patients, and two or more were applied in 40%. Microbiological diagnosis was achieved in 99 (50%) patients, 69 (70%) by only one method (42% FBAS, 23% EAT, 15% sputum, 9% BAL, 7% blood culture, and 4% urinary antigen). Seventy-eight (39%) patients underwent a fiberoptic-bronchoscopy when not receiving mechanical ventilation. Higher rates of microbiological diagnosis were observed in the invasive group (56 vs. 39%, *p* = 0.018). Patients with microbiological diagnosis more frequently presented changes in their empirical antibiotic scheme, mainly de-escalation.

**Conclusions:**

A comprehensive approach might be undertaken for microbiological diagnosis in critically ill nonventilated HAP. Sputum sampling determined one third of microbiological diagnosis in HAP patients who were not subsequently intubated. Invasive methods were associated with higher rates of microbiological diagnosis.

**Electronic supplementary material:**

The online version of this article (10.1186/s13054-019-2348-2) contains supplementary material, which is available to authorized users.

## Introduction

Hospital-acquired pneumonia (HAP) is a frequent event during an intensive care unit (ICU) stay and is characterized by a pneumonia acquired during hospitalization, in patients without invasive mechanical ventilation [1–3]. Despite improved prevention measures, antimicrobial therapy, and supportive care, it remains an important cause of morbidity and mortality [1–3]. HAP is the leading cause of death among hospital-acquired infections, with estimates of its associated mortality ranging from 20 to 50% [2, 4–6].

Microbiological diagnosis is fundamental for guiding HAP treatment, allowing a targeted, effective antibiotic choice, and reducing the associated impact of ineffective empiric antibiotic regimens or the unnecessary use of broad-spectrum antibiotics [1]. Yet the current understanding of HAP pathogens is based mainly on data derived from ventilator-associated pneumonia (VAP) [7–15]. Although some studies have reported the pathogens in HAP that occur outside the ICU [16–18], there is no systematic description of the diagnostic approaches that should be used to efficiently obtain an microbiological diagnosis in HAP, mainly when critically ill [1].

In comparison with VAP, HAP poses additional challenges for respiratory sampling and, thus, for obtaining microbiological diagnosis. The utility of sputum cultures or distal sampling for HAP has not been comprehensively evaluated [1]. The recent guidelines for HAP/VAP recognized that for some patients, whom non-invasively sampling is not possible, invasive sampling is an option [1, 3]; however, the literature is scarce to support one method over the other in HAP. In this study, we aimed to describe the diagnostic approaches used in a cohort of HAP acquired during an ICU stay and their associated clinical impact.

## Materials and methods

### Study population

We conducted a retrospective analysis of a prospective cohort including patients from six medical and surgical ICUs at an 800-bed teaching hospital in Spain. Patients older than 18 years admitted to these ICUs for 48 h or more with clinical suspicion of HAP or VAP were prospectively and consecutively included. Patients with severe immunosuppression (neutropenia after chemotherapy or hematopoietic transplant, drug-induced immune suppression in solid-organ transplant or cytotoxic therapy, and HIV-infected patients) were excluded. The institution’s internal review board approved the study (Comite Etic d’Investigacio Clinica, registry number 2009/5427), and written informed consent was obtained from patients or their next of kin.

### Definition of pneumonia

Clinical suspicion of pneumonia was based on clinical criteria as suggested in the guidelines [1, 5, 19]: (1) new or progressive radiologic pulmonary infiltrate, (2) together with at least two of the following: temperature > 38 °C or < 36 °C, leukocytosis > 12,000/mm^3^ or leukopenia < 4000/mm^3^, or purulent respiratory secretions. HAP was defined in patients who developed pneumonia after 48 h of hospitalization when not receiving invasive mechanical ventilation (iMV) [1, 20].

### Data collection

All relevant data were collected upon ICU admission and at the onset of pneumonia from the medical records and bedside flow charts, including clinical, laboratory, radiological, and microbiological information. Patients’ follow-up was extended to death, to hospital discharge, or up to 90 days after the diagnosis of pneumonia. The assessment of severity included the Acute Physiology and Chronic Health Evaluation (APACHE)-II [21] and the Sequential Organ Failure Assessment (SOFA) score [22], calculated upon ICU admission and at HAP diagnosis.

### Microbiologic assessment and methods

We tried to assess all patients upon clinical diagnosis of HAP, aiming to establish a microbiological diagnosis. Lower respiratory airway samples that could be collected for quantitative bacterial and fungal cultures were (1) sputum, (2) endotracheal aspirate (EAT), (3) bronchial aspirate through a fiberoptic-bronchoscopy (FBAS), and (4) bronchoalveolar lavage (BAL), blinded or through a fiberoptic-bronchoscopy. Only samples of sputum or tracheal aspirates of high quality (i.e. < 10 squamous cells and > 25 leukocytes per optical microscopy field) were processed for culture. Additionally, blood cultures (recommended to all patients) and pleural fluid (if a pleural puncture was indicated) could be collected, as well as urinary antigens of *Legionella* sp. and *Streptococcus pneumoniae* (mainly recommended for early-onset HAP). Pathogen identification and susceptibility testing were performed by standard methods [23]. Microbiological diagnosis was defined by the presence of at least one potentially pathogenic microorganism (PPM) in respiratory samples above predefined thresholds (sputum, EAT, FBAS > 10^5^ colony-forming units/mL or BAL > 10^4^, or any count if the patient was receiving a new systemic antibiotic treatment). Blood cultures were considered positive if an alternative cause of bacteremia was ruled out [23].

Polymicrobial pneumonia was defined when more than one PPM was identified as causative agents. The initial empiric antimicrobial treatment was chosen following a local adaptation of the 2005 ATS/IDSA guidelines [5], based on the most frequently isolated pathogens and their patterns of antimicrobial sensitivity at our institution. The empiric antimicrobial treatment was considered appropriate when the isolated pathogens were susceptible in vitro to at least one of the antimicrobials administered. Multi-drug-resistant pathogens were defined based on consensus definition [24].

Antibiotic de-escalation was considered when physicians changed the antibiotic regimen to a narrower spectrum regimen, stopped the coverage for a class of pathogens (e.g., *Staphylococcus aureus*), or reduced the number of antibiotics prescribed [25–27]. Escalation was considered when physicians introduced a new regimen with broader coverage. We further divided the patients whom the empiric antibiotic scheme was maintained in those that no change was done, and in those whom an additional antibiotic was added to the empiric regimen.

### Statistical analysis

To analyze the diagnostic yield of the sampling method, we divided HAP patients into those who were subsequently intubated and those who were not, since in patients under iMV, the airway is easy to reach for lower respiratory sampling collection. We also compared patients who received a fiberoptic-bronchoscopy when undergoing or not undergoing iMV.

Data were presented as numbers (proportions) and as means ± SD or medians [p25-p75]. Qualitative or categorical variables were compared with the chi-square test or Fisher’s exact test, as appropriate. Quantitative continuous variables were compared using the unpaired Student *t* test, one-way ANOVA, and Mann-Whitney or Kruskal Wallis tests as appropriate. All tests were two-sided, and Stata 13.1 was used for all analyses.

## Results

Of the 488 patients enrolled during the cohort period, we excluded 288 (59%) patients who were diagnosed with pneumonia while receiving mechanical ventilation (i.e., VAP). Therefore, we analyzed 200 (41%) patients with HAP.

### Patient characteristics

**Fig. 1:**
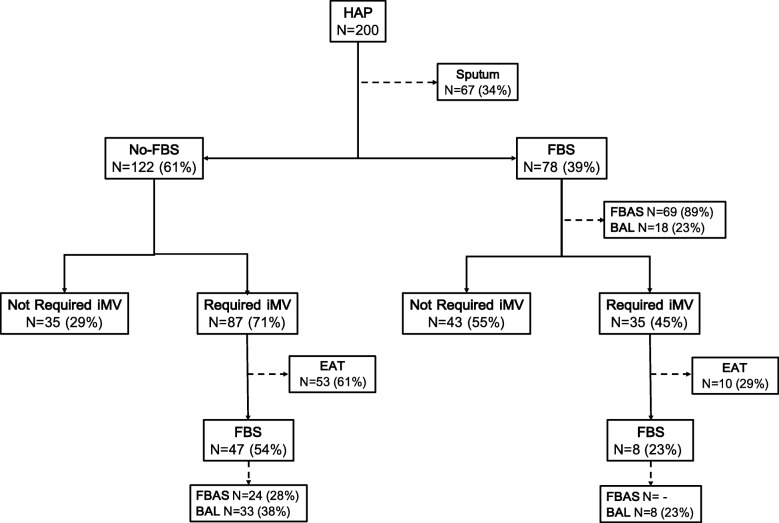
Time flow-chart for the microbiological assessments performed in 200 patients with hospital-acquired pneumonia. BAL bronchoalveolar lavage, EAT endotracheal aspirate, FBS fiberoptic-bronchoscopy, FBAS fiberoptic-bronchoscopy aspirate, HAP hospital-acquired pneumonia, iMV invasive mechanical ventilation

The main clinical characteristics upon ICU admission and at onset of HAP are shown in Table 1. Mean age was 66 years, and there was a high proportion of males. Approximately one third had a chronic comorbidity. The main cause of ICU admission was acute respiratory failure followed by shock and postoperative status. One hundred twenty-two patients (61%) required iMV after the onset of HAP (Fig. 1), and 72 (59%) intubations occurred within 24 h of diagnosis. The median ICU length of stay was 13 [7–26] days, and 85 (43%) patients died in the hospital. Patients who needed iMV after HAP diagnosis presented higher hospital mortality than those who did not [62 (51%) vs. 23 (30%), *p* = 0.003].

**Table 1:** General characteristics of patients with hospital-acquired pneumonia (HAP)

Variable	Entire cohort (*n* = 200)
Age (year), mean SD	66 ± 12
Sex (male), *n* (%)	137 (69%)
Smoker (current or past), *n* (%)	105 (53%)
Alcohol abuse (current or past), *n* (%)	52 (26%)
Comorbid Conditions, *n* (%)	
Chronic heart disease	76 (38%)
Chronic lung disease	73 (37%)
Solid cancer	55 (28%)
Diabetes	48 (24%)
Chronic hepatic disease	48 (24%)
Chronic renal failure	24 (12%)
Severity at ICU admission	
APACHE II at ICU admission, mean SD	15.8 ± 6
SOFA at ICU admission, median [p25-p75]	6 [4–9]
Reason of ICU admission, *n* (%)	
Hypoxemic respiratory failure	57 (29%)
Hypercapnic respiratory failure	32 (16%)
Shock	28 (14%)
Postoperative	42 (21%)
Non-surgical abdominal condition	15 (7%)
Altered mental status	8 (4%)
Multiple trauma	3 (2%)
Other	15 (7%)
Severity Scores at diagnosis	
APACHE II Score at HAP, mean SD	16.3 ± 5
SOFA Score at HAP, median [p25-p75]	6 [4–9]
Features at diagnosis	
Temperature, mean SD	36.6 ± 1
Leukocytes, mean SD	14,495 ± 7224
PaO_2_/FiO_2_, mean SD	178 ± 79
Bilateral infiltrates, *n* (%)	66 (33%)
Multilobar infiltrates, *n* (%)	109 (55%)
Pleural effusion, n (%)	86 (44%)
ARDS at pneumonia diagnosis, *n* (%)	27 (14%)
Previous use of antibiotic, *n* (%)*	151 (76%)
Outcomes	
ICU length-of-stay, days median [p25-p75]	13 [7–26]
Hospital length-of-stay, days median [p25-p75]	37 [22–61]
ICU mortality, *n* (%)	62 (31%)

*ARDS* Acute respiratory distress syndrome, *APACHE II* Acute Physiology and Chronic Health Evaluation, *SOFA score* Sequential Organ Failure Assessment score

*Not necessarily concomitant to sample collection

### Diagnostic approach

**Fig. 2:**
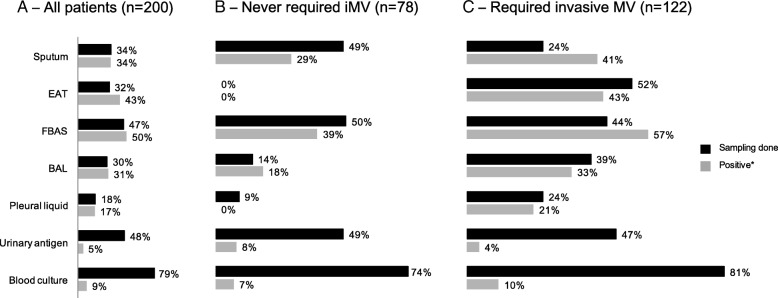
Sampling methods and corresponding positivity in **a** whole cohort, **b** patients not requiring invasive mechanical ventilation, and **c** patients requiring invasive mechanical ventilation. BAL bronchoalveolar lavage, EAT endotracheal aspirate, FBAS fiberoptic-bronchoscopy aspirate, iMV invasive mechanical ventilation. *Percentage among those in whom the method was performed

In the 200 patients with HAP, 89% underwent at least two methods for microbiological assessment (median 3 [2–4] methods). Patients who required iMV had a higher number of microbiological assessments than those who did not (3 [2–4] vs. 2 [2, 3], *p* < 0.001, respectively). Respiratory samples were obtained in 186 (93%) patients, and at least two respiratory methods were applied in 40%. Blood cultures (79%), urinary antigen (48%), and FBAS (47%) were the methods most commonly applied to microbiological assessment (Fig. 2, Table 2, and Additional file 1: Table S1). Sputum and BAL were performed in almost one third of patients, while 18% had pleural liquid cultures. Sputum, EAT, FBAS, and BAL were the methods that obtained the highest proportions of positivity (Fig. 2, Table 2, and Additional file 1: Table S1), followed by pleural liquid, blood culture, and urinary antigen testing.

**Table 2:** Pathogens and contribution of different methods to microbiological diagnosis

	Entire cohort (*n* = 200)	Never received invasive MV after HAP (*n* = 78)	Received invasive MV after HAP (*n* = 122)	*P* value
Definitive causative pathogen	99 (50%)	31 (40%)	68 (56%)	0.027
Gram negative non-fermenting bacteria	39/99 (39%)	11/31 (35%)	28/68 (41%)	0.59
*Pseudomonas aeruginosa*	30/99 (30%)	10/31 (32%)	20/68 (29%)	0.78
*S. aureus*	24/99 (24%)	6/31 (19%)	18/68 (27%)	0.44
MSSA	15/99 (15%)	4/31 (13%)	11/68 (16%)	0.77
MRSA	9/99 (9%)	2/31 (7%)	7/68 (10%)	0.72
Gram negative enteric bacteria	24/99 (24%)	8/31 (26%)	16/68 (24%)	0.81
Community pathogens (*S. pneumococcus, Haemophylus influenzae*)	9/99 (9%)	3/31 (10%)	6/68 (9%)	> 0.99
Virus	3/99 (3%)	–	3/68 (4%)	0.55
Other	9/99 (9%)	1/31 (3%)	8/68 (12%)	0.27
Polymicrobial	17/99 (17%)	3/31 (10%)	14/68 (21%)	0.182
Multi-drug resistant	40/99 (40%)	12/31 (39%)	28/68 (41%)	0.82
Microbiological diagnosis by				
Sputum	23/99 (23%)	11/31 (36%)	12/68 (18%)	0.051
EAT	27/99 (27%)	–	27/68 (40%)	< 0.001
FBAS	46/99 (47%)	15/31 (48%)	31/68 (46%)	0.80
BAL	18/99 (18%)	2/31 (7%)	16/68 (24%)	0.041
Pleural fluid	6/99 (6%)	–	6/68 (9%)	0.051
Urinary antigen	5/99 (5%)	3/31 (10%)	2/68 (3%)	0.175
Blood culture	14/99 (14%)	4/31 (13%)	10/68 (15%)	> 0.99
Microbiological diagnosis by				
1 method	69/99 (70%)	27/31 (87%)	42/68 (62%)	0.015
2 methods	20/99 (20%)	4/31 (13%)	16/68 (23%)	
3 methods	10/99 (10%)	–	10/68 (15%)	
Microbiological diagnosis uniquely defined by 1 method				
Sputum	10/69 (15%)	9/27 (33%)	1/42 (2%)	0.001
EAT	16/69 (23%)	–	16/42 (38%)	< 0.001
FBAS	29/69 (42%)	12/27 (44%)	17/42 (41%)	0.81
BAL	6/69 (9%)	–	6/42 (14%)	0.075
Pleural fluid	–	–	–	–
Urinary antigen	3/69 (4%)	3/27 (11%)	–	0.056
Blood culture	5/69 (7%)	3/27 (11%)	2/42 (5%)	0.37

*BAL* bronchoalveolar lavage, *EAT* endotracheal aspirate, *FBS* fiberoptic bronchoscopy, *FBAS* fiberoptic-bronchoscopy aspirate, *HAP* hospital-acquired pneumonia, *iMV* invasive mechanical ventilation

**Fig. 3:**
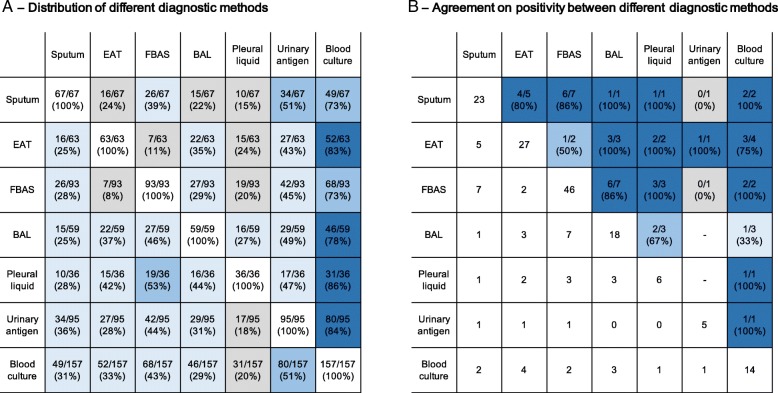
Distribution and agreement of different sampling methods (**a** cross-tabulation of different methods; **b** agreement on the same pathogen when both methods were positive). Square colors divided as dark blue for agreement ≥ 75%, blue for agreement between ≥ 50 and < 75%, light blue for agreement between ≥ 25 and < 50%, and grey for agreement < 25%. BAL bronchoalveolar lavage, EAT endotracheal aspirate, FBAS fiberoptic-bronchoscopy aspirate

Microbiological diagnosis was possible in 99 (50%) patients. Patients who required iMV had a higher proportion of microbiological diagnosis than those who did not (56 vs. 40%, *P* = 0.027, Table 2). Thirty-eight (19%) patients received a new antibiotic before sample collection and had a lower proportion of microbiological diagnosis than those who did not (34 vs. 53%, *p* = 0.036). Overall, the most common pathogens identified were Gram-negative non-fermenting bacteria (39/99, 39%), followed by *Staphylococcus aureus* (24/99, 24%) and Gram-negative enteric bacteria (24/99, 24%). The prevalence of polymicrobial HAP was 17% (17/99), while 40% had a MDR pathogen. The distribution of causative pathogens was similar in those who required iMV and those who did not (Table 2). The cross-tabulation of different methods for microbiological assessment and their agreement on the same pathogen, when positive, are shown in Fig. 3. The average overall agreement was 80% (40/50). Indeed, there was 85% agreement for sputum with other respiratory samples (11/13), 80% for EAT (8/10), 81% for FBAS (13/16), and 91% for BAL (10/11).

The majority of microbiological diagnoses were determined by only one method (69/99, 70%), with differences among those who required iMV and those who did not (*p* = 0.015). FBAS was the only method responsible for the diagnosis of 42% (29/69) patients, followed by EAT (23%), sputum (15%), BAL (9%), and blood culture (7%).

One hundred twenty-five (63%) patients underwent invasive sampling, of whom 78 (39%) were applied fiberoptic-bronchoscopy while not receiving iMV (Fig. 1). Patients who required iMV after invasive sampling were more severe at HAP diagnosis (Additional file 2: Table S2). There was no significant difference in the proportion of final microbiological diagnoses when stratifying by fiberoptic-bronchoscopy when receiving or not receiving iMV (*p* = 0.112); however, among the patients who did not require iMV, the rate of microbiological diagnosis was 10% higher (95% CI, − 12 to 32%) in those who underwent fiberoptic-bronchoscopy. When stratifying patients according to non-invasive (sputum and EAT) or invasive (FBAS and BAL) respiratory methods, we observed higher proportions of microbiological diagnoses in those who underwent at least one invasive method (56 vs. 39%, risk difference 17%, 95% CI, 3–31%, *p* = 0.018), mainly due to those who required iMV.

### Antibiotic management and duration

The majority of patients received the initial antibiotic regimen in accordance with the 2005 ATS/IDSA guidelines; empiric antibiotic treatment was adequate in 71% (70/99 patients) (Table 3). Patients who had a microbiological diagnosis more frequently changed their empirical antibiotic regimen (*P* = 0.006), driven by de-escalation (30 vs. 8%). However, patients who had a microbiological diagnosis also received longer total antibiotics duration than patients without microbiological diagnosis, although similar duration when considered only the empiric antibiotic scheme.

**Table 3:** Antibiotic management and duration among those patients who had microbiological diagnosis or not

	Whole cohort		*P* value^a^	Never required iMV		Required iMV		*P* value^b^
	No microbiological diagnosis (*n* = 101)	Microbiological diagnosis (*n* = 99)		No microbiological diagnosis (*n* = 47)	Microbiological diagnosis (*n* = 31)	No microbiological diagnosis (*n* = 54)	Microbiological diagnosis (*n* = 68)	
Antibiotic treatment								
ATS guideline adherence	71 (70%)	21 (79%)	0.168	32 (68%)	26 (84%)	39 (72%)	52 (77%)	0.43
Adequate empiric treatment		70/99 (71%)			22/31 (71%)		48/58 (71%)	0.97
Change on empiric treatment	49 (49%)	67 (68%)	0.006	18 (38%)	16 (52%)	31 (57%)	51 (75%)	0.001
De-escalation	8 (8%)	30 (30%)	< 0.001	3 (6%)	5 (16%)	5 (9%)	25 (36%)	< 0.001
Continued empiric	60 (59%)	39 (40%)		31 (66%)	19 (61%)	29 (54%)	20 (29%)	
Continued empiric + add new antibiotic	23 (23%)	16 (16%)		11 (23%)	5 (16%)	12 (22%)	11 (16%)	
Escalation	10 (10%)	14 (14%)		2 (4%)	2 (6%)	8 (15%)	12 (18%)	
Empiric treatment duration, median [p25-p75]	9 [6–10]	7 [4–11]	0.197	9 [7–11]	8 [6–12]	7 [5–10]	7 [4–11]	0.066
Total treatment duration, median [p25-p75]	10 [7–15]	14 [10–22]	0.004	10 [8–14]	13 [9–22]	11 [7–16]	14 [10–22]	0.036

*ATS* American Thoracic Society, *iMV* invasive mechanical ventilation

^a^Comparison between those with and without microbiological diagnosis

^b^Overall comparison between four groups

## Discussion

We could achieve microbiological diagnosis in 50% of 200 patients with HAP occurring during ICU stay using an intensive diagnostic approach. Upon HAP clinical diagnosis, around 40% of patients underwent fiberoptic-bronchoscopy while not receiving iMV. Finally, invasive respiratory sampling was associated with a higher rate of microbiological diagnosis.

Recent recommendations from the FDA recognized that there are three different types of nosocomial pneumonia with different all-cause mortality rates: non-ventilated HAP, ventilated HAP, and VAP [28, 29]. Interestingly, the highest mortality has been observed in patients with HAP who subsequently required iMV. In a recent summary of these recommendations, Talbot highlighted the necessity to have information about sampling and causative pathogens in the non-VAP population [28]. Our study is the first one to provide this information in a detailed way, which can be very useful for empirical treatment adequacy and for future RCT studying new antibiotics.

Being able to achieve a microbiological diagnosis in HAP has important consequences for patient care. First, it can support the suspicion of infection in a new lung infiltrate appearing concomitantly with fever in a critically ill patient, a frequent challenge for the attending physician [30]. Second, it makes possible to target the empiric antibiotic scheme more accurately, thus increasing the likelihood of clinical cure, preventing the selection pressure to further resistances, and reducing costs and unnecessary side effects [1]. Our findings corroborated two important phenomena reported elsewhere: (1) patients with microbiological diagnosis more commonly had an adaptation in their empiric antibiotic regimen and (2) patients without microbiological diagnosis received shorter total antibiotic treatment [31, 32]. Although a microbiological diagnosis is central in all infections both for epidemiological studies and for bedside care by clinicians, it becomes fundamental for hospital-acquired infections, because of higher probability of resistant pathogens, greater amount of antibiotic use and side effects, and associated costs.

Interestingly, one third of patients underwent sputum collection, which was positive in 34% of cases after ensuring sample quality and performing quantitative cultures. Very few data are available on the applicability of sputum in HAP [1, 16, 17]. In our experience, this non-invasive diagnostic method should be encouraged, as it already is for community-acquired pneumonia [33]. Indeed, when only one diagnostic method was positive, 15% of microbiologic confirmations were due to sputum, and in patients who were not subsequently intubated, this proportion was even higher (33%). Despite the limited numbers of patients allowing for pair-wise comparisons between methods, we observed a good agreement on retrieving the same pathogen (80% on average). As expected it was higher for invasive methods (FBAS vs. BAL, 86% of agreement). In our protocol, we tried to obtain as much as possible respiratory samples to increase the likelihood of identifying a causative pathogen, and the good agreement observed is reassuring. When two methods were discordant, respecting the sample quality check and cutoff values, clinicians interpreted the episode as polymicrobial and treated both pathogens, which is sound in critically ill patients. Taking different respiratory samples also increases the risk of false positives (i.e., colonization). We could not evaluate the actual impact that discordance between methods would have in clinician’s decision in a scenario where there would be a hierarchy between methods, for instance.

In this observational study, patients assessed with an invasive diagnostic method had higher rates of microbiological diagnosis. Although there is evidence that invasive and non-invasive approaches have a comparable impact on patient-centered outcomes in VAP [1], no evidence is available for HAP in immunocompetent patients [1]. In fact, the 2016 IDSA/ATS guidelines propose non-invasive respiratory sampling in HAP, although the panel agreed that there may be factors that prompt clinicians to consider invasive sampling [1]. In a small single-center randomized trial aiming to compare invasive and non-invasive approaches in patients with HAP outside the ICU, Herer et al. found that clinical cure rates at 28 days were similar between groups; however, the study was rather exploratory in nature, with several limitations and a small sample size [18].

Because of the barriers to obtaining lower respiratory tract samples in HAP, we cannot straightforwardly extrapolate the evidence from VAP to HAP. Indeed, an invasive approach might have higher clinical utility in HAP, particularly in those patients who will not require iMV. A key point when discussing invasive vs. non-invasive tactics in HAP is the feasibility and safety of performing a fiberoptic-bronchoscopy. Several reports show that fiberoptic-bronchoscopy, followed by BAL or mini-BAL, can be conducted in patients with acute respiratory failure and community- and healthcare-acquired pneumonia and is even safer when non-invasive ventilation and high-flow oxygen therapy are applied [34–39]. In a landmark trial, Azoulay et al. showed that an invasive approach had a similar rate of intubation to a non-invasive approach in non-ventilated, immunosuppressed patients with acute respiratory failure [40].

Invasive mechanical ventilation after HAP diagnosis was commonly needed in our population of critically ill patients, being applied 60% of the time within 24 h. Despite its clear implications for prognosis, having an endotracheal tube vastly facilitates access to a lower respiratory tract sample using either invasive or non-invasive approaches. The ability to predict which patients will need iMV in the next hours can help guide clinicians faced with the decision of performing a prompt fiberoptic-bronchoscopy or postponing it until after the intubation. The development of a prediction tool is beyond the scope of this study, but we observed that severity, hypoxemia, and chest X-ray patterns were associated with intubation after performing a fiberoptic-bronchoscopy.

Our study has some strengths. We included prospective cases of HAP acquired during an ICU stay from six ICUs. Our center also has a comprehensive clinical decision-making protocol for achieving microbiological diagnosis in lung infections, which means that our data are relevant for the description of microbiological diagnosis in HAP. Moreover, the causative pathogens responsible for HAP in our cohort are similar to those reported elsewhere, where Gram-negative bacteria have been implicated in 55% to 85% of HAP cases and Gram-positive cocci (particularly *Staphylococcus aureus*) account for 20% to 30% [7, 9–11, 13, 14, 41], thus increasing the generalizability of our results. In addition, the results of this study cover an unmet need of knowledge (microbiological diagnosis of HAP) highlighted by the recent IDSA ATS and International guidelines for HAP and VAP [1, 3].

However, there are several limitations that must be highlighted. First, our study is retrospective and single-center and, although we collected data from six ICUs with different profiles (from general medical to respiratory and liver units), the single-center characteristic decreases the generalizability of our findings. Second, our study is observational and allowed us for a reliable description of real-life diagnostic methods approaches for achieving microbiological diagnosis in HAP, our primary objective. However, the crude associations found for the potential benefit of invasive methods are exploratory and not causal; a well-designed, controlled randomized trial is now warranted to define the management of HAP regarding the use of invasive or non-invasive methods. Third, we could recruit 200 patients, which limited our ability to explore subgroups and pair-wise comparisons between different methods, but to the best of our knowledge, this is one of the first and largest studies reporting all these different diagnostic methods in critically ill nonventilated HAP [42]. Third, we could not achieve 100% of respiratory samples in the cohort; however, we believe that 93% represents a very high proportion of patients, considering the daily care in an ICU. Fourth, our population comprised critically ill patients, who commonly require iMV, and our results may not be applicable to patients outside the ICU. Fifth, we did not have a “gold standard” to confirm that the pathogen identified was responsible for the infection and not only colonizing the airways, a potential limitation particularly for sputum cultures. To limit the number of false positives, we used the most standard quality assessment to accept only lower airway representative samples. Sixth, at the time the current study was conducted, our center did not have routine access to rapid diagnostic methods because they were not standard of care, but these methods have been shown to be promising tools for pathogen identification in HAP [43]. The performance of rapid diagnostic methods in nonventilated HAP, utilizing different sampling strategies, must be evaluated and could produce different results compared to our findings. Particularly, rapid diagnostic methods could increase the sensitivity for pathogen identification in those patients already receiving a new antibiotic upon sample collection, a fact that might explain the reason we achieved only 50% of microbiological diagnosis using traditional culture methods [44]. Finally, we did not conduct a cost-effective analysis [1, 18], which is a key element when comparing different respiratory sampling methods.

## Conclusion

In summary, our study raises the point that a comprehensive approach might be undertaken for microbiological diagnosis in critically ill nonventilated HAP. Sputum determined one third of microbiological diagnosis in HAP patients who were not subsequently intubated. Invasive methods were associated with higher rates of microbiological diagnosis; however, this might be replicated in other populations and through a randomized, well-designed, controlled trial.

## Additional files

Additional file 1: **Table S1.** Methods for diagnostic approach. Additional data about diagnostic methods stratified by patients with hospital-acquired pneumonia who required or not mechanical ventilation. (PDF 132 kb)

Additional file 2: **Table S2.** Comparison between patients who did invasive sampling while receiving or not receiving invasive mechanical ventilation. Additional data about general characteristics and microbiological diagnosis between patients who did invasive sampling methods while receiving or not mechanical ventilation. (PDF 143 kb)

## Abbreviations

APACHE II: Acute Physiology and Chronic Health Evaluation II
ATS/IDSA: American Thoracic Society/Infectious Disease Society of America
BAL: Bronchoalveolar lavage
EAT: Endotracheal aspirate
FBAS: Fiberoptic-bronchoscopy aspirate
HAP: Hospital-acquired pneumonia
HIV: Human immunodeficiency virus infection
ICU: Intensive care unit
iMV: Invasive mechanical ventilation
MDR: Multi-drug resistant
PPM: Potentially pathogenic microorganism
SOFA: Sequential Organ Failure Assessment
VAP: Ventilator-associated pneumonia
