# Supplementary material for: Invasive and non-invasive diagnostic approaches for microbiological diagnosis of hospital-acquired pneumonia
Source: Crit Care. 2019 Feb 18;23:51. doi: 10.1186/s13054-019-2348-2 (PMC6379979; doi:10.1186/s13054-019-2348-2)
Supplement: Supplementary file 1 — Table S1. Methods for diagnostic approach. Additional data about diagnostic methods stratified by patients with hospital-acquired pneumonia who required or not mechanical ventilation. (PDF 132 kb) [file 13054_2019_2348_MOESM1_ESM.pdf]

Table S1. Methods for diagnostic approach

	Entire cohort (n=200)	Never received invasive MV after ICUAP (n=78)	Received invasive MV after ICUAP (n=122)	P value
**Respiratory tract assessment**				
Sputum sampling done	67 (34%)	38 (49%)	29 (24%)	<0.001
Sputum culture positive	23 (12%)	11 (14%)	12 (10%)	0.36
Sputum culture positive among those with sputum culture done	23/67 (34%)	11/38 (29%)	12/29 (41%)	0.29
EAT sampling done	63 (32%)	-	63 (52%)	
EAT culture positive	27 (14%)	-	27 (22%)	
EAT culture positive among those with EAT culture done	27/63 (43%)	-	27/63 (43%)	
FBAS sampling done	93 (47%)	39 (50%)	54 (44%)	0.43
FBAS culture positive	46 (23%)	15 (19%)	31 (25%)	0.31
FBAS culture positive among those with FBAS culture done	46/93 (50%)	15/39 (39%)	31/54 (57%)	0.071
BAL sampling done	59 (30%)	11 (14%)	48 (39%)	<0.001
BAL culture positive	18 (9%)	2 (3%)	16 (13%)	0.011
BAL culture positive among those with BAL culture done	18/59 (31%)	2/11 (18%)	16/48 (33%)	0.48
Pleural liquid sampling done	36 (18%)	7 (9%)	29 (24%)	0.008
Pleural liquid positive	6 (3%)	-	6 (5%)	0.083
Pleural liquid positive among those with liquid pleural done	6/36 (17%)	-	6/29 (21%)	0.32
Urinary antigen				
Urinary antigen investigation*	95 (48%)	38 (49%)	57 (47%)	0.78
Positive urinary antigen	5 (3%)	3 (4%)	2 (2%)	0.38
Positive urinary antigen among those with urinary investigation done	5/95 (5%)	3/38 (8%)	2/57 (4%)	0.39
Blood culture				
Blood culture done	157 (79%)	58 (74%)	99 (81%)	0.25
Positive blood culture	14 (7%)	4 (5%)	10 (8%)	0.41
Positive blood culture among those with blood culture done	14/157 (9%)	4/58 (7%)	10/99 (10%)	0.50

* S. pneumoniae and Legionella
