# Supplementary material for: Invasive and non-invasive diagnostic approaches for microbiological diagnosis of hospital-acquired pneumonia
Source: Crit Care. 2019 Feb 18;23:51. doi: 10.1186/s13054-019-2348-2 (PMC6379979; doi:10.1186/s13054-019-2348-2)
Supplement: Supplementary file 2 — Table S2. Comparison between patients who did invasive sampling while receiving or not receiving invasive mechanical ventilation. Additional data about general characteristics and microbiological diagnosis between patients who did invasive sampling methods while receiving or not mechanical ventilation. (PDF 143 kb) [file 13054_2019_2348_MOESM2_ESM.pdf]

Table S2. Comparison between patients who did invasive sampling while receiving or not receiving invasive mechanical ventilation

	Not fiberoptic-bronchoscopy*		Fiberoptic-bronchoscopy*		
	**Not Required iMV (n=35)**	**Required iMV (n=87)**	**Not Required iMV (n=43)**	**Required iMV (n=35)**	**P value**
Age, years , mean SD	66 ± 13	66 ± 12	66 ± 14	67 ± 11	0.99
APACHE II Score at HAP diagnosis, mean SD	15.2 ± 4	17.7 ± 6	13.3 ± 4	17.5 ± 5	<0.001
SOFA Score at HAP diagnosis, median [p25-p75]	5 [4-7]	7 [5-10]	5 [4-8]	6 [4-8]	<0.001
PaO_2/FiO_2, mean SD	192 ± 93	169 ± 71	209 ± 86	151 ± 63	0.006
Bilateral infiltrates, n (%)	9 (26%)	36 (41%)	6 (14%)	15 (43%)	0.007
Multilobar infiltrates, n (%)	16 (46%)	51 (59%)	22 (51%)	20 (57%)	0.58
ARDS at pneumonia diagnosis, n (%)	2 (6%)	17 (20%)	0 (0%)	8 (24%)	0.003
**Respiratory support**					
NIV previous of HAP diagnosis	4 (11%)	17 (20%)	14 (33%)	11 (31%)	0.077
NIV to provide support upon HAP diagnosis	9 (26%)	23 (26%)	10 (23%)	12 (34%)	0.73
Need of invasive mechanical ventilation					<0.001
Within <24h		60 (69%)		12 (34%)	
Within 24-72h		17 (20%)		13 (37%)	
Within >72h		10 (11%)		20 (29%)	
Time to intubation, days					0.001
Mean SD		0.91 (2)		1.57 (2)	
Median [p25-p75]		0 [0-1]		1 [0-3]	
**Definitive causative pathogen**	12 (34%)	47 (54%)	19 (44%)	21 (60%)	0.112

ARDS: Acute respiratory distress syndrome, APACHE II: Acute Physiology and Chronic Health Evaluation, FBS: fiberoptic-bronchoscopy, HAP: hospital-acquired pneumonia, iMV: invasive mechanical ventilation, NIV: non-invasive ventilation, SOFA score: Sequential Organ Failure Assessment.

* In this table, only considered the invasive approaches (fiberoptic-bronchoscopy) performed before invasive mechanical ventilation in those patients who were subsequently intubated.
